# Gel Characteristics and Digestion of Composite Protein Emulsion-Filled Gels with Varying Soy and Whey Protein Ratios in the Matrix

**DOI:** 10.3390/gels12010037

**Published:** 2025-12-31

**Authors:** Qiuyan Liu, Georgina Benewaa Yeboah, Sen Wang, Haowei Zhang, Juan Wu, Qingling Wang, Yu Cheng

**Affiliations:** 1 School of Food and Biological Engineering, Jiangsu University, Zhenjiang 212013, China; 2212318016@stmail.ujs.edu.cn (Q.L.); ama.benewaa@yahoo.com (G.B.Y.); 3220905019@stmail.ujs.edu.cn (S.W.); 2212018052@stmail.ujs.edu.cn (H.Z.); wujuan@ujs.edu.cn (J.W.); 2 Department of Biochemistry and Biotechnology, Kwame Nkrumah University of Science and Technology, University Post Office, Kumasi GPS AK-448-4944, Ghana; 3 Institute of Food Physical Processing, Jiangsu University, Zhenjiang 212013, China; 4 College of Food Science and Engineering, Yangzhou University, Yangzhou 225127, China; qlw@yzu.edu.cn

**Keywords:** emulsion-filled gel, dual protein, gel matrix, digestion

## Abstract

The effect of mixed soy and whey protein in the matrix on properties and digestion characteristics of emulsion-filled gels was investigated. Different matrix protein concentrations (8–14%) with a composite soy and whey protein (SW) ratio of 5:5 were screened using gel hardness. The better-performing gel (13%) was selected for matrix composition studies. Soy and whey composite protein mixed at different ratios (S/W = 0/10, 3/7, 5/5, 7/3, and 10/0) was dispersed into another soy-whey (S/W = 6/4) composite emulsion and gelled thermally. Different hybrid protein ratios in the matrix can alter the textural and rheological properties and, consequently, the digestion kinetics of mixed plant-animal gel systems. The storage modulus was highest at an S/W ratio of 0/10. The hardness of gel with the S/W ratio matrix of 0/10 was 3.10 and 9.60 times higher than that of 5/5 and 10/0 (*p* < 0.05). The SW ratio did not affect water-holding capacity or springiness (*p* > 0.05). All the gels had swelling ability below 10% except SW 10/0 (around 60%). Gels with an S/W of 5/5 exhibited a lower hydrolysis degree and rate during gastric digestion, while the reverse occurred during intestinal digestion. The compact gel network might limit pepsin’s accessibility to cleavage sites.

## 1. Introduction

Emulsion-filled protein gels are protein gels that contain emulsified droplets [1]. These types of emulsion gels are suitable as carrier systems for both fat-soluble and water-soluble bioactive compounds [1,2]. Thus, they can be used to develop novel food products [3,4,5,6]. The presence of these oil-coated droplets improves the gel network and increases the strength of the protein gels by serving as active fillers [7]. The properties or type of the emulsifier, emulsifier concentration, the filler concentration and size, and processing conditions can influence the properties of the emulsion-filled gels [1,4,8,9,10,11]. The protein emulsifiers that coat the oil droplets interact with other proteins in the gel matrix, thereby affecting the gel strength [12,13]. The matrix composed of mono-proteins has been the focus of most work on emulsion-filled protein gels [1]. By contrast, emulsion-filled protein gels with a matrix consisting of dual proteins remain largely unexplored.

The combined use of plant and animal proteins has recently garnered significant attention in food applications as the world transitions towards eco-friendly, economical, and sustainable production [3,6,14,15,16]. Thus, the combined use of soy and whey proteins in gel formulations has been studied [2,17,18,19,20,21]. These studies indicated that mixing these proteins could improve the properties of the plant proteins. Increasing the soy protein ratio resulted in gels with reduced strength and hardness but enhanced water-holding capacity compared to those made from a single protein. However, the fabrication of emulsion-filled gels using soy and whey protein blends has not been fully explored.

Therefore, the characteristics of heat-set emulsion gels prepared from emulsions containing composite soy-whey proteins at various ratios were investigated in our previous study [22]. Both proteins were observed to coexist at the interface of the emulsified oil droplets and within the gel matrices. It was interesting to learn about the roles of soy and whey protein compositions at the interface and in the matrix in modulating properties and digestion of emulsion protein gel. However, it is not easy to determine the soy-whey protein ratio at the interface and in the matrix. Therefore, we conducted a two-step process to elucidate the role of soy-whey protein ratios in the filler and matrix. Hybrid animal and plant proteins partitioning at the oil-water interface have been shown to modulate the properties of protein emulsion gels [12,23,24,25]. Moreover, the effect of the interfacial compositions made up of hybrid soy and whey protein at varying proportions on the characteristics and digestion of whey protein gel was established [13]. Fillers containing hybrid soy-whey proteins in various proportions can produce emulsion-filled gels with enhanced and varied properties, including hardness and springiness, swelling capacity, microstructure, and digestion kinetics. Moreover, the structure and composition of the gel matrix have been demonstrated to affect the mechanical properties [12,23,24,26,27] and the digestion of gel [19,28,29,30,31]. However, the role of soy and whey protein proportions in the gel matrix on gel properties and digestion was not explored.

Although studies on soy-whey composite protein gels exist, there is limited reporting on the effect of soy-whey composite protein gels filled with oil droplets emulsified using a soy-whey protein mixture. In this study, we developed emulsion-filled gels with a soy and whey protein mixture at the interface and continuous phase. Different protein concentrations in the matrix were screened, and the one that yielded the best texture and gel strength was selected for the matrix study. The aim was to investigate the effects of a gel matrix (mixed soy and whey protein) fabricated with varying protein proportions on the properties, microstructure, and digestion characteristics of a soy and whey hybrid protein emulsion-filled gel.

## 2. Results and Discussion

### 2.1. Effect of Protein Concentration on the Mechanical Properties of Emulsion-Filled Protein Gels

To select a working concentration for the gelled system and to evaluate the gel matrix, the protein ratio in the fillers was kept constant (S/W = 6/4), and the total protein concentration of the gel matrix varied. The whey-to-soy protein ratio in the matrix was set at 5/5.

#### 2.1.1. The Physical Morphology of the Hybrid Protein Gel Filled with Emulsion

**Figure 1:**
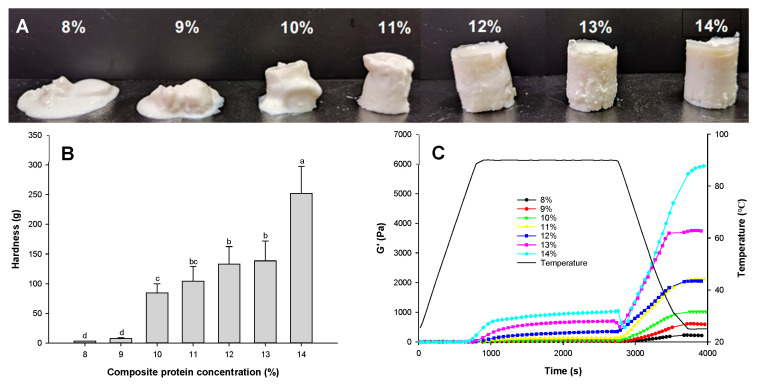
Gel appearance (**A**), hardness (**B**), and storage modulus G (**C**) of emulsion-filled gels with a matrix of hybrid soy and whey protein at a ratio of 5/5 and different total protein concentrations. Means with different lowercase letters (a–d) differ significantly (*p* < 0.05).

The total protein concentration in the matrix can influence gel formation. It can be seen (Figure 1A) that increasing the total protein concentration resulted in the formation of stable, self-supporting gel structures. At total protein concentrations below 10% (8% and 9%), the emulsified gels were paste-like. They were unable to form a self-supporting gel structure. The reason was that low protein concentration may result in low protein-molecule density. The protein molecules were insufficiently dense within the matrix, resulting in considerable spacing between them. It can weaken molecular interactions because intermolecular forces are inversely proportional to the distance between molecules. Fewer physical crosslinking interactions between protein molecules resulted in loose gel networks, leading to weak gel strength [7,32]. At a 10% total protein concentration, the gel could support itself, although it displayed a cylindrical shape with significant deformation. It may be due to the WPI and SPI protein concentrations in the gel reaching their lowest gelling concentrations. Moreover, the gels with a total protein concentration of 11–14% contained sufficient protein molecules, thereby reducing the distance between them. It can enhance molecular interactions and produce self-supported gels without deformation [7,11]. It can therefore be concluded that protein interactions within the gel contributed to the formation of the gel network and structure at these ratios [12].

#### 2.1.2. Effect of Composite Protein Concentration on Hardness and Storage Modulus of Hybrid Protein Gel Filled with Emulsion

Gel hardness increased with increasing total protein concentration (*p* < 0.05), exhibiting a maximum value at a total protein concentration of 14% (Figure 1B). When total protein concentration was raised from 9% to 10%, gel hardness sharply increased by 10.44 times (*p* < 0.05). Further increasing total protein concentration from 10% to 13% and 14%, the gel hardness rose by 0.64 and 1.99 times, respectively (*p* < 0.05). The gel hardness test results confirmed the gel’s appearance and indicated that a total protein concentration of 10% was the lowest gelling concentration. This observation is consistent with other similar studies [26,27,28], which found that increasing protein concentration improved the hardness of composite gels. Thus, varying the concentration of mixed protein emulsion gels can produce gels with different textural properties for diverse food applications.

A gel hardness test was performed using compression to assess the gels’ resistance to deformation. The recovery of gel deformation was not estimated. The storage modulus during gel formation was determined to evaluate gel elasticity, as shown in Figure 1C. The emulsion gels had very low rigidity and strength under lower total protein concentrations (8%, 9%), as evidenced by the lower G’. It confirms their gel morphology (Figure 1A). Gel rigidity increased with increasing protein concentration [18], which supports the results of hardness (Figure 1B). The steady rise in G’ during the cooling stage suggests that the hydrogen bonds were involved in strengthening the gel network [22].

Based on gel morphology, hardness, and rheological data, the emulsion gels prepared with a mixed protein concentration of 13% were selected for the study on the gel matrix, aiming to investigate the impact of matrix composition on emulsion gel characteristics and digestion kinetics.

### 2.2. Effect of Gel Matrix Composition on Gel Properties

#### 2.2.1. Rheological Dynamics

**Figure 2:**
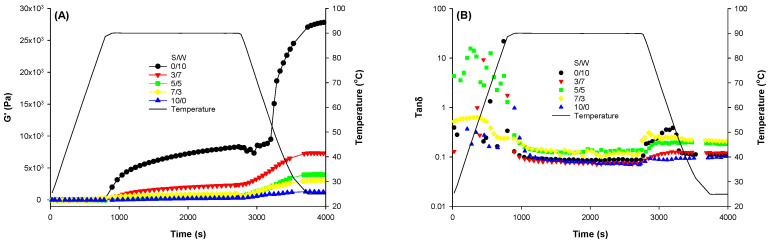
The storage modulus (**A**) and tan delta (**B**) of the emulsion-filled gels with a matrix of hybrid soy and whey protein at different ratios.

Figure 2A is a display of the temperature cycle of the mixed protein emulsified droplets embedded into composite protein gels prepared at different ratios of soy protein to whey protein (S/W). The onset of gelation (Figure 2A) in the mixed systems was similar, likely because the denaturation of both WPI and 7S of SPI occurs at approximately the same temperature (75 °C) [17]. There was a steady, relatively higher rise in the storage modulus during the heating phase, a finding similar to that observed for composite soy and protein gel [17,18] and composite soy and protein emulsion gel [13,22]. During the holding phase (90 °C), there was an increase in the storage modulus (G’) of all samples. It can be attributed to protein denaturation, which exposes bonds used for intramolecular protein aggregation [7,11]. This phase was more pronounced for the matrix with S/W of 0/10. This may be due to the emulsion-filled whey protein gel (S/W = 0/10) exhibiting a low gelation temperature. Moreover, whey protein is more soluble than soy protein, thereby enhancing protein-protein interactions. The oil droplets in composite protein emulsions functioned as active fillers and enhanced interactions among whey protein molecules. In the other emulsion-filled gels with a matrix with S/W of 5/5, 7/3, and 10/0, there was a similar trend, as shown in Figure 2A, indicating similarity in low gel strength and gelation kinetics [18]. The soy protein usually exhibited low solubility and formed large protein aggregates, hindering interactions between whey protein molecules and between whey protein and dissolved soy protein [22]. The CLSM results below confirm the presence of large protein aggregates.

The cooling phase led to further gel strengthening, as evidenced by higher G’ values across all samples. The increased G’ during the cooling phase has been attributed to the formation of bonds, particularly hydrogen bonds [33]. At the end of the cycle, the gel at S/W 0/10 had the highest G’ value, followed by 3/7, with 10/0 being the lowest. This was not surprising, as it was consistent with the gel-solubility results in 8M urea (Table 1). The gel’s solubility in 8 M urea indicates the strength of hydrogen bonds [33]. The gel with the whey protein matrix (S/W of 0/10) displayed the highest solubility in 8 M urea. The incorporation of SPI aggregates into the gel network also contributed to the strengthening of G’ values [18,22]. All gels exhibited elastic behavior, as tan δ (Figure 2B) was less than 1.

The final storage modulus of gels with the protein mixture (S/W 3/7, 5/5, and 7/3) as the matrix decreased significantly with increasing soy protein ratio. This trend is similar to that reported by Jose et al. (2016) [17], who found that the denaturation temperatures of both WPI and 7S of SPI are identical. However, the denaturation temperature of 11S in SPI is higher (94 °C). Since the heating temperature during gel formation is 90 °C, both proteins (WPI and 7S of SPI) can be involved in the formation of the gel network in the matrix by aggregation. At the same time, 11S of SPI did not unfold and was incorporated into the gels as particulate fillers [22]. According to McCann et al. (2018) [18] and Roesch & Corredig (2005) [34], in the soy-whey gel system, WPI is the main component responsible for gel network formation. These findings suggest that both protein components actively contributed to the rigidity and, consequently, the formation and strengthening of the composite emulsion gels [13,22]. This has been evident from our previous work on whey-soy hybrid protein emulsion gels [22]. Increasing the SPI ratio in the mixed protein matrix resulted in weaker gels, as indicated by the G’ values. Consequently, it confirms the role of the WPI in forming the gel network. Our results were consistent with studies on the mixture of whey and other plant proteins [23,35,36].

**Figure 3:**
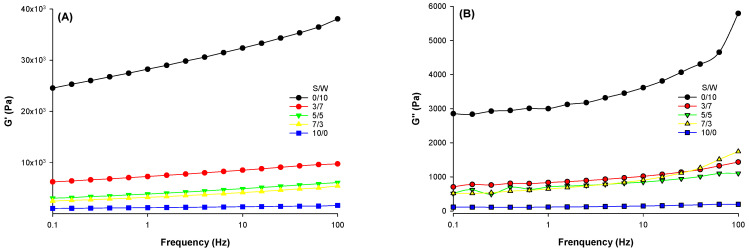
Frequency sweep ((**A**)-storage modulus; (**B**)-loss modulus) of the emulsion-filled protein gels with a matrix of hybrid soy and whey protein at different ratios.

The trend observed for G’ and G’’ (Figure 3) indicated that all gels exhibited elastic behavior as the storage modulus (G’) was higher than the loss modulus (G’’). With increasing frequency, the shearing time decreases, resulting in higher stored energy within the polymer [37]. The increasing frequency with an associated rise in G’ indicated that the gels were of weaker rigidity and the network was maintained by hydrogen, disulfide, and non-covalent bonds [38]. Based on the rheological results, it can be concluded that, for these mixed protein matrices, SPI content/ratio is key to producing gels with varied strengths.

#### 2.2.2. Textural Properties

**Figure 4:**
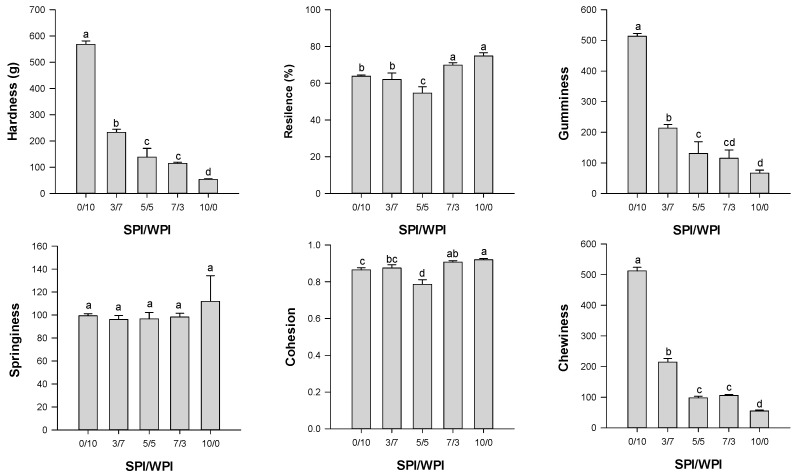
Texture profile analysis of the emulsion-filled gels with a matrix of hybrid soy and whey protein at different ratios. Means with different lowercase letters (a–d) differ significantly (*p* < 0.05).

Texture is a property that affects the acceptability of food products. Figure 4 is a representation of the texture profile analysis of emulsion-filled gels with a matrix of hybrid soy and whey protein at different ratios. The effect of matrix protein ratios on gel hardness is consistent with the results of G’. Hardness decreased with increasing SPI ratio (*p* < 0.05), with the highest value at an S/W ratio of 0/10 and the lowest at 10/0. The hardness of gel with the matrix of S/W ratio of 0/10 was 3.10 and 9.60 times higher than that of 5/5 and 10/0 (*p* < 0.05). This indicates the active role of whey protein in the formation and strengthening of the gel [13,18,39]. The observed increase in hardness with increasing WPI content is consistent with previous studies [22,34]. The reason may be the stronger hydrogen bonding in gels with a whey protein matrix, as indicated by their solubility in urea (Table 1). The results suggest that hydrophobic and covalent interactions occurred between WPI and SPI during gelation [18]. This is demonstrated by the gel’s solubility in SDS and β-ME, as shown in Table 1. Gels with an S/W matrix of 5/5 exhibited 5.80- and 1.92-fold higher solubility in SDS than those with 10/0 and 0/10, respectively. On the other hand, the lower gel hardness in SPI-dominant emulsion-filled gels could be due to glycinin (11S)’s little contribution to the gel network, as we described above.

Moreover, beta-conglycinin (7S) lacks disulfide bonds, preventing it from facilitating gelation via disulfide bonds. This is evident from the gel’s solubility in β-ME. Gels with a protein mixture (S/W 3/7, 5/5, and 7/3) as the matrix exhibited lower solubility in β-ME when compared to the gels with the matrix of soy protein (S/W 10/0) (*p* < 0.05). However, due to its lower denaturation point (66–79 °C), 7S may have been denatured and participated in gelation [40,41], playing a limited role in improving the gel network. SPI has been reported to exhibit this behavior when heated at neutral pH [40]. The weaker gels were due to the limiting effect of beta-glycinin (7S) on the coaggregation of glycinin (11S) [40]. Thus, the SPI-dominated mixed gels formed larger aggregates, limiting their incorporation into the gel network and thereby affecting gel hardness [22]. It was evident from the CLSM images below.

Gumminess and chewiness followed the pattern of hardness. Gel with the matrix of S/W 10/0 indicated 2.92- and 6.69-fold greater gumminess than that of 5/5 and 10/0, respectively (*p* < 0.05). Similarly, the gel with the S/W 10/0 matrix showed 4.23- and 8.33-fold greater chewiness than the 5/5 and 10/0 matrices, respectively (*p* < 0.05). More rigid gels are generally gummier and chewier, as confirmed by other studies [35,42,43]. There were significant differences in resilience and cohesion among the hybrid gels (*p* < 0.05). Except for S/W 5/5, all the mixed protein emulsion gels had a resilience above 60%, indicating that the matrix composition affected gel properties. Gels with the high-soy-protein matrices S/W 7/3 and 10/0 showed 0.28- and 0.37-fold greater resilience than 5/5, respectively. For springiness, there was little difference among the protein ratios in the matrix (*p* > 0.05). This supports the findings on texture, which indicate that SW 10/0 was the softest gel. It thus had a greater ability to bounce back to its original height than the more rigid gels, which crumble under pressure. S/W of 10/0 and 5/5 were the most cohesive and least cohesive gels, respectively (*p* < 0.05). The differences in textural properties may be due to the antagonistic or synergistic effects of the plant-animal protein interaction on the structure and network of the mixed protein emulsion gel [12,23,24].

The results indicate that adjusting the matrix composition, specifically varying the ratios of plant to animal protein, in emulsion gelled systems can provide foods with diverse textural properties. This can be used to overcome the limitation of textural properties commonly observed in plant protein gelled food systems [12,23,24].

#### 2.2.3. WHC and Swelling Capacity

**Figure 5:**
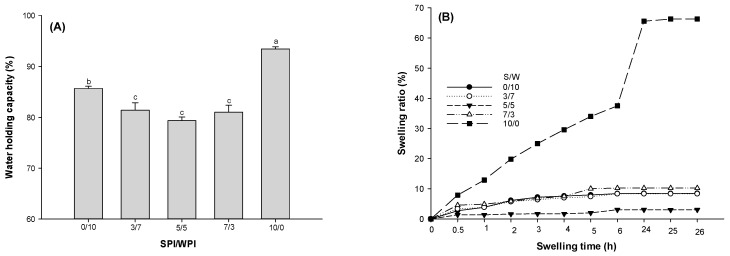
Water holding (**A**) and swelling capacities (**B**) of the emulsion-filled gels with a matrix of hybrid soy and whey protein at different ratios. Means with different lowercase letters (a–c) differ significantly (*p* < 0.05).

As shown in Figure 5A, WHC increased 9.06% when the gel matrix changed from S/W of 10/0 to S/W of 0/10 (*p* < 0.05). Increasing SPI in mixed soy-whey systems has been shown to increase WHC [17]. However, for the gel with a mixed-protein matrix, the SPI-to-WPI ratio did not affect WHC (*p* > 0.05). The WHC of gel with the matrix of S/W 5/5 is 85.44% and 93.18% of gel with S/W 10/0 and 0/10 (*p* < 0.05). The reason may be phase separation in the gel microstructure of the mixed protein system [19]. Soy protein aggregates form via hydrophobic interactions among hydrophobic residues, thereby exposing hydrophilic residues. The hydrophilic residues can bind water molecules, leading to improved WHC. Gels with higher rigidity retain water within their networks more effectively than those with lower rigidity [44,45]. The weaker gel strengths of the mixed protein systems might have accounted for this pattern. The reduced WHC of the mixed systems also indicates weaker interactions between the proteins and water molecules within these systems [46].

The emulsion-filled gels in this study had higher WHC than those reported by Cui et al. (2020) [47], who studied SW composite gels under US pretreatment at different times. They noted that WPI had the highest WHC, while SPI had the lowest. It was probably because they used mixed-protein gels, whereas this study incorporated oil droplets into the gel network. These differences can also be ascribed to the solvent used in the emulsion gel preparation (mixed protein emulsion), the higher protein concentrations, and the plant-to-animal protein ratios used in this study. The active filling effect of soy and whey composite protein emulsified oil droplets could improve the gel network [7]. The better gel network may exhibit higher physical binding capacity for water.

Swelling capacity was determined in the mixed protein emulsion gels for 26 h (Figure 5B). The pattern was similar to that of the WHC. S/W of 5/5 had the least swelling capacity, while S/W 10/0 had the highest. For S/W of 10/0, swelling capacity exceeded 30% after 4 h and reached 60% by the end of the study. All other gels had less than 10% swelling, which levelled off after 4 h, indicating saturation. This suggests that, although the matrix composition affected the swelling ratio, the effect was not significant except at an S/W ratio of 10/0. The reduced swelling capacity of the mixed gels may be due to a weak gel structure and entrapped water within the gel network during gel formulation [48]. Thus, the formulation of mixed protein emulsion gels with proteins embedded in the structure could serve as food systems for controlled or delayed release, as they might limit the access of digestive enzymes/juices to cleavage sites.

#### 2.2.4. Structural Force Analysis of Composite Protein Gel Filled with Emulsion

Gel solubility, which determines the chemical bonds responsible for gel strengthening, was measured in four solvents (Table 1). Increasing the SPI ratio increased the disulfide bond and reduced hydrogen bonds. All the mixed protein emulsion-filled gels were highly soluble in β-ME solution, demonstrating that disulfide bonds mainly sustained the heat-induced mixed protein emulsion-filled gels. This finding aligns with other studies [25,49]. Increasing the SP ratio promoted solubilization in β-ME solutions, whereas reducing it promoted solubilization in urea solutions (*p* < 0.05). Non-covalent bonds maximized at SW 5/5, after which they declined with increasing SPI (*p* < 0.05). The results show that mainly disulfide bonds, along with some non-covalent and hydrogen bonds, maintained the structure of the mixed protein emulsion-filled gels. Nonetheless, the S/W of 5/5 had almost identical proportions of non-covalent and disulfide bonds, which may have accounted for its weak mechanical properties, as indicated by the gel texture.

#### 2.2.5. Microstructure of Gel Matrix Made of Composite Soy-Whey Protein

**Figure 6:**
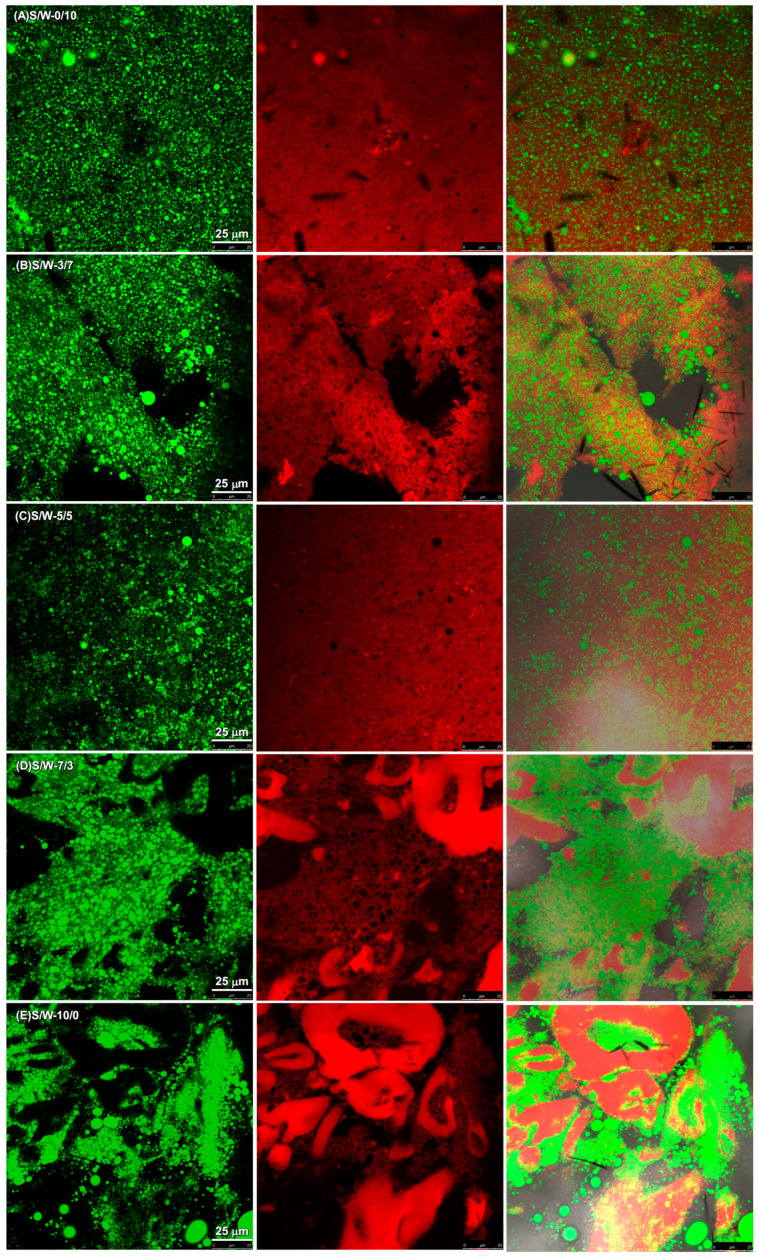
Confocal laser scanning microscopy of the emulsion-filled gels with a matrix of hybrid soy and whey protein at different ratios. Green and red color represent oil and protein, respectively.

**Figure 7:**
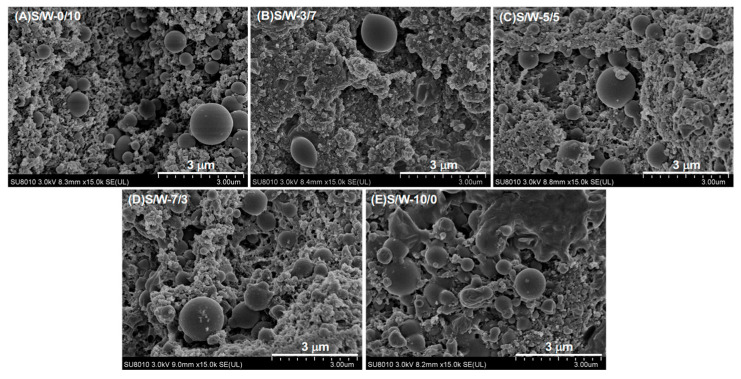
Scanning electron microscope of the emulsion-filled gels with a matrix of hybrid soy and whey protein at different ratios.

The microstructure of the emulsion-filled composite soy and whey protein gels with the matrix at different soy-whey ratios is assessed using CLSM and SEM, as shown in Figure 6 and Figure 7. The SEM and the CSLM are consistent.

In the CLSM images, green and red represent oil and protein, respectively. The combined images showed that green oil droplets filled the red protein network. When comparing the protein channel images, the whey protein matrix exhibited a dense microstructure. The whey protein network was not readily apparent under CLSM due to its size. It has exceeded the limits of optical microscopy. Therefore, SEM was performed to visualize the gel network at the mesoscopic scale. The SEM images confirm the presence of oil droplets within a protein network in the gel samples. The whey protein matrix demonstrated a dense, 3D porous network composed of small protein aggregates. By contrast, the soy protein matrix exhibited a coarse and concentrated protein network under CLSM. It consisted of the SEM result. The soy protein matrix exhibited a network composed of concentrated protein and aggregates lacking porosity. CSLM results suggested that gels with the S/W 5/5 matrix were predominantly composed of a whey protein network, whereas those with the S/W 7/3 matrix were predominantly composed of a soy protein network. This was evident in SEM. Moreover, gels with the S/W 3/7 matrix contained both networks, indicating phase separation in those samples.

Gels with the S/W 0/10 and 5/5 matrix displayed a more uniform and denser microstructure than other gel samples. Moreover, they contained smaller protein aggregates. A uniform and dense microstructure can resist gel deformation, leading to higher hardness in the texture [3,19,26]. It is evident from the hardness of the gels with the S/W 0/10 matrix (Figure 4), whereas that of the S/W 5/5 matrix denied it. This was not surprising, as protein concentration can significantly affect gel texture [3,33]. Whey protein was a predominant contributor to the gel network in the S/W 0/10, 7/3, and 5/5 matrices. A decrease in whey protein content could reduce hardness. The larger aggregates were observed in gels with S/W matrices of 7/3 and 10/0. The large aggregates observed in the SEM images of gels with the S/W10/0 matrix (Figure 7E) were consistent with the CLSM results. The formation of larger aggregates may be due to intermolecular disulfide bonds of undenatured 11S [40]. They could not form a rigid texture and did not incorporate into the gel network. They could not provide sufficient support to the gel structure, thereby reducing the protein content in the gel network and resulting in low hardness. The coarse network of those gel samples also resulted in low hardness.

In addition to the gel matrix, the filler can affect the gel properties of the emulsion-filled gel. All the gel samples were prepared with the same emulsions. It was supposed to display a similar oil droplet size. However, it was interesting that the size of the oil droplets varied across the gel samples, as shown in the CLSM oil channel images. It was found that filler size in the whey protein predominant network was smaller than that in the soy protein predominant network. In emulsion-filled gels with matrices of 0/10 and 5/5, the emulsified oil droplets exhibited little flocculation and remained dispersed, as confirmed by SEM. By contrast, in emulsion-filled gels with matrices of 7/3 and 10/0, the emulsified oil droplets flocculated and were concentrated in CSLM images and confirmed by SEM. The larger oil droplet size in the soy protein predominant gel indicated that coalescence occurred during gel formation. It might be due to the high porosity of the soy-protein-dominant network. The large porosity could hold several oil droplets. Limited space might drive the oil droplet close. The oil droplets might flocculate and rupture the interfacial membrane, leading to coalescence. Coalescence increased the radius of oil droplets, thereby reducing the Laplace pressure. The filler’s resistance to deformation decreased, resulting in lower hardness. This implies that there were weaker protein interactions between the matrix components and between the filler and the matrix. Poor gel interactions with increasing SPI can explain the decrease in gel hardness.

### 2.3. Simulated Digestion of Mixed Protein Matrix

**Figure 8:**
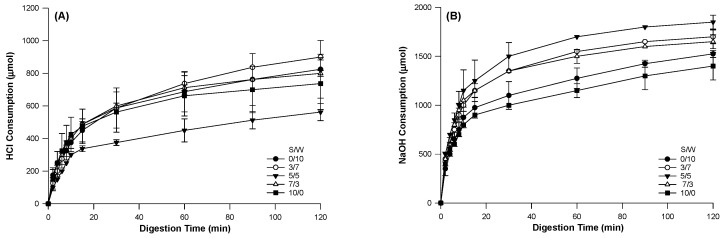
Acid (HCl) and base (NaOH) consumption during gastric (**A**) and intestinal digestion (**B**), respectively, of the emulsion-filled gels with a matrix of hybrid soy and whey protein at different ratios.

Studying the digestion behavior of the developed food systems is vital for their adoption and applications. The amount of acid and base consumption during gastric and intestinal digestion for the composite protein emulsion gels is displayed in Figure 8. Enzymatic hydrolysis of protein can expose amino and carboxyl groups, which require the consumption of HCl and NaOH to maintain a constant pH. Therefore, the consumption of HCl and NaOH is mainly due to protein hydrolysis. The HCl and NaOH consumption can indicate the exposure content of amino and carboxyl groups, suggesting the hydrolysis rate. The gels exhibited different digestion behaviors in HCl (Figure 8A) and NaOH (Figure 8B). The highest acid consumption was observed in S/W 3/7, followed by S/W 0/10 (the hardest gel) and S/W 5/5 (the least). Though the S/W of 3/7 was not the hardest, it had larger droplets than the S/W of 0/10, coupled with a lower swelling ratio. The larger droplet sizes might also have resulted in higher HCl consumption, as aggregate size influences hydrolysis [50]. Acid consumption for the S/W 10:0 gel was lower, suggesting a slower digestion rate [22,25], possibly because it was the softest, with the highest swelling and WHC. The softer nature and higher swelling capacity allowed for the infusion of pepsin, and the gel required less acid [51]. S/W of 5/5 was the gel with the least consumption of acid. This gel had the lowest swelling ratio, was soft, and was and the least rigid. The uniform distribution of proteins and droplets, together with their soft, denser structure, might have limited the diffusion of pepsin and facilitated hydrolysis.

In the intestinal phase, the single matrix gels used the least NaOH, with an S/W of 10/0. The softer gel matrix and larger pore sizes (resulting from the microstructure) of the S/W 10/0 might have contributed to this [13,37,52]. The gels with mixed soy and whey ratios exhibited higher base consumption, indicating a higher rate of pancreatin reaction and diffusion. The S/W of 5/5 with equal ratios of soy and whey in the network had the highest base consumption.

Since the digestion pattern was not evident, the acid and base consumption data from gastric and intestinal digestion were fitted to a biphasic model. The reason for selecting a biphasic model was that we hypothesized that gel digestion comprises two stages [53,54]. At the first stage, enzymes hydrolyze the substrates in the digestive fluid and diffuse to the gel surface. In the second stage, enzymes diffuse into the gel network and bind to their substrates. When the amount of HCl used was fitted to the biphasic model to study pepsin diffusion and protein hydrolysis of these polymers (Table 2), it was observed that increasing SPI generally increased the rate of reaction, k, during the first step of the biphasic model. This indicates that at higher SPI gel matrix ratios, pepsin diffused more, leading to greater protein hydrolysis. This might be because increasing the SPI ratio decreased gel strength and hardness, and softer gels with higher swelling capacity allow the diffusion of enzymes for breakdown. The rate of pepsin diffusion and protein hydrolysis was in the order S/W 10/0 > 3/7 > 7/3 > 5/5 > 0/10. SW 10:0 had the highest swelling capacity, facilitating the diffusion of pepsin into the matrix and promoting the breakdown of the soy-whey gel.

The WPI dominant gel (S/W of 0/10) had the least amount of HCl consumption (A) and the rate of reaction (k) during the first step of the first kinetic order reaction. The hard, compact nature and smaller pore sizes of this gel limited pepsin’s accessibility to the cleavage sites. This decreased the acid concentration in the medium, thereby reducing the reaction rate. Except for the S/W of 10/0, all the other gels exhibited significantly greater HCl consumption and slower reaction rates (k) as gastric digestion progressed into the subsequent first-order-second phase. S/W of 5/5 had the highest acid consumption, possibly due to weaker bonds between the matrix and the fillers, which facilitated hydrolysis.

During the intestinal phase, gels showed greater diffusion and protein hydrolysis as SPI increased, thereby increasing the reaction rate. This might be explained by the softer nature of the gels, which facilitates the diffusion of pancreatin and promotes hydrolysis. The second step of this model indicated that S/W of 0/10 had the highest consumption of the base, with a lower reaction rate, suggesting delayed hydrolysis, as observed in the gastric phase.

Thus, in these composite soy and whey gels, the WPI-dominant gel, S/W 0/10, had the least degree of hydrolysis both in gastric and intestinal digestion. These models indicate that modifying the matrix composition by combining plant and animal proteins can produce gels with distinct digestion kinetics and release profiles, thereby enabling programmable release of bioactive compounds at specific time points during digestion.

## 3. Conclusions

This study investigated the impact of a mixed soy-whey protein gel matrix on the physicochemical properties and digestion behavior of soy-whey protein emulsion-filled gels. The results indicate that the soy-to-whey ratio in the matrix exerted distinct effects on the microstructure and mechanical properties of the soy-whey composite gels. The thermally induced gelation of the mixed proteins yielded varying gel structures and properties, with the two proteins contributing differently to the gel’s mechanical properties. The mixed protein gels were intermediate between the single-protein emulsion gels in terms of gel storage modulus, texture, and WHC. A higher WPI ratio produced gels with better properties, and the emulsion-filled soy protein gel had weak mechanical properties. In simulated digestion, increasing WPI decreased pepsin diffusivity. It increased the reaction rate in the first phase of gastric digestion, though the rate was reduced in the second phase of the biphasic model. This delay in protein hydrolysis is due to the compact nature and lower swelling ability of the WPI-dominant gels compared to the SPI-dominant gels. In the intestinal stage, gels showed greater enzyme diffusion and protein hydrolysis, with increased SPI. The reaction rate and hydrolysis rate were slower for SW of 0/10.

Thus, varying protein concentrations and using plant-animal protein matrices at different ratios can be considered for application in the food industry as the world moves towards more sustainable production and raw material acquisition for food security and environmentally friendly production. In the future, studies on the digestion behavior of bioactive substances embedded in these gelled systems should be conducted to assess their feasibility for controlled release and as carriers for bioactive compounds. Other gelation methods can also be considered with plant-animal proteins. Moreover, the static in vitro digestion model used does not account for gastric emptying, pH changes, and mechanical breakdown during digestion. The limitations of the current model led to deviations from in vivo conditions. The semi-dynamic and dynamic in vitro digestion model can be considered in future work.

## 4. Materials and Methods

### 4.1. Materials

Whey protein isolate (WPI) (90.90 wt%) was a product of Hilmar Company (Hilmar, CA, USA). Soy protein isolate (SPI) (90.0 wt%) was donated by Shandong Yuwang Ecological Food Industry Co., Ltd. (Yucheng, China). Soybean oil was purchased from a local supermarket without further processing. Pepsin (250 U/mg), pancreatin (the trypsin activity of 35 U/mg), and β-mercaptoethanol were purchased from Sigma-Aldrich (St. Louis, MO, USA). All other chemical reagents were of analytical grade.

### 4.2. Preparation of Whey-Soy Protein Composite Emulsion

The SPI (S) and WPI (W) were mixed at a 6:4 (S/W, *w*/*w*) ratio, and hybrid protein solutions with a final protein concentration of 10 mg/mL (pH 7.0) were prepared. The SPI and WPI ratio was selected based on our previous results [13]. Its emulsion-filled whey protein gel exhibited balanced gel properties and a high whey protein alternative content. The composite protein solution was mixed with soybean oil at a 9/1 (*w*/*w*) ratio by homogenization for 1 min in an HG-15A high-speed blender (DAIHAN Scientific Co., Ltd., Wonju-si, Korea) at 10,000 r/min. The pre-emulsions were subjected to an AH-BASIC high-pressure homogenizer (ATS Engineering Inc., Toronto, ON, Canada) and passed through twice at 300/50 bar to prepare the fine emulsions. Emulsions were kept at 4 °C until further use.

### 4.3. Determination of the Protein Concentration for the Gels

The composite SW protein gels were prepared at a 5:5 (S/W, *w*/*w*) protein ratio at different total pure protein concentrations (8–14%, *w*/*v*). The composite protein mixture (of different concentrations) was dispersed in the SW (6/4) composite emulsion (Section 2.2), which served as the solvent. The composite protein emulsions were poured into containers, sealed, and heated in a water bath at 90 °C for 30 min. Tubes were then cooled to room temperature (RT) and stored at 4 °C overnight. Gels were equilibrated to room temperature for 1 h before use.

### 4.4. Dynamic Rheological Measurement

Temperature-cycling tests were performed using a TA DHR-1 hybrid rheometer (TA Instruments, New Castle, UK) using parallel plate geometry (40 mm), as described by Mao et al. (2021) [25]. The strain of 0.5% (within the linear viscoelastic region) and the oscillation frequency of 1 Hz were used. The samples were heated from 25 to 90 °C at 5 °C/min, then incubated at 90 °C for 30 min, and finally cooled to 25 °C at 5 °C/min. Afterward, frequency oscillation was conducted on the gels at 25 °C. The frequency and strain were 1 Hz and 0.5%, respectively.

### 4.5. Texture Profile Analysis (TPA) of Gels

The measurement was conducted using a Texture Analyzer (TA-XT Plus, Stable Microsystems, Surrey, UK) with an aluminum cylindrical probe of 50 mm diameter. A test speed of 1 mm/s, compression strain of 25%, and force of 3 g were used [55]. At least six replicates were taken for each treatment.

### 4.6. Preparation of Soy-Whey Protein Emulsion Droplets Embedded into Soy-Whey Protein Gel

SPI-WPI composite protein (13% *w*/*v*) mixed at different ratios (SW 0/10, 3/7, 5/5, 7/3, and 10/0) was dispersed into the SW 6/4 composite emulsion (Section 2.2) (as solvent) for 30 min using a magnetic stirrer. Sodium chloride (50 mmol/L) was added to emulsions, followed by sampling into tubes. The containers were sealed and heated to 90 °C in a water bath for 30 min. Afterward, the samples were cooled to RT and held at 4 °C overnight. Rheology and texture analysis were completed on the gels (Section 4.6) according to Section 4.4 and Section 4.5, respectively.

### 4.7. Water Holding Capacity (WHC) of Gels

The WHC was performed after Yeboah et al. (2025) [13]. The percentage of differences in the weights indicated the WHC of the gels. Water holding capacity (%) = ((W_0_ − W_t_)/W_0_) × 100%, where W_0_ is the gel weight before centrifugation, and W_t_ is the gel weight after centrifugation.

### 4.8. Swelling Capacity of Gels

For the swelling ratio, the procedure of Yeboah et al. (2025) [13] was followed. It was indicated by the percentage difference between the gel’s weight at the initial time and at a specific time (t).

### 4.9. Gel Solubility

Referring to the procedure of Yeboah et al. (2025) [13], four solvents were formed, including phosphate buffer (PB, 50 mmol/L, pH 7.0), 8 mol/L urea in PB, 0.5% (*w*/*v*) sodium dodecyl sulfate in PB, and 0.25% (*w*/*v*) 2-mercaptoethanol in PB. Half-gram ground gel samples were suspended in solvents (4.5 mL) and blended, then heated at 80 °C for 30 min. The supernatant from the mixture was collected after centrifugation at 5000× *g* for 15 min. The biuret method was used to determine the protein content in different supernatants. The forces present in the gels were demonstrated by the solubility of proteins in various solvents.

### 4.10. Confocal Laser Scanning Microscopy (CLSM)

Gel samples (1 mm thick) were stained with 10 µL Fast Green and 20 µL Nile Red. The stained gel was placed on a concave slide and covered with a slip. Images were obtained at a 40× magnification using Leica TCS SP5.

### 4.11. Scanning Electron Microscopy (SEM)

Cube gel samples were subjected to 2.5% glutaraldehyde for 4 h [13]. Afterward, they were rinsed three times in PB (10 mmol/L pH 7.0), followed by dehydration in a graded ethanol series (30–100% (*v*/*v*)) for 10 min at each concentration. The dehydrated gels were dried using a critical point dryer (CPD-300, Leica Mikrosysteme Vertrieb GmbH, Wetzlar, Germany). The microstructure images of dry gel samples were captured using a scanning electron microscope after the gels were coated with gold.

### 4.12. Simulated Digestion and Degree of Hydrolysis

The procedure of Brodkorb et al. (2019) [56] was performed with slight modifications, as described by Zhang et al. (2023) [57]. Ground gels were blended with simulated saliva fluid (SSF, pH 7.0) at a 50:50 proportion without the addition of amylase. The bolus was formed by stirring at 37 °C for 2 min, then mixed with simulated gastric fluid (SGF) at a 50:50 ratio. The pH was adjusted to 3.0 with 1 mol/L hydrochloric acid. CaCl_2_ (0.15 mmol/L) and pepsin (2000 U/mL) were then added to the mixture. The amount of acid added to maintain pH 3 during gastric digestion was recorded. Pepsin digestion was halted by enhancing the pH to 7 with sodium hydroxide (1 M) after 120 min. The gastric chyme was filtered through a sieve with a 1.4 mm pore size. The chyme that passed the sieve was used for intestinal digestion. It was blended with simulated intestinal fluid (SIF) at an equal ratio, followed by the addition of bile salts (10 mmol/L) and CaCl_2_ (0.6 mmol/L). Afterward, trypsin (100 U/mL) was added. The amount of base required to maintain a pH of 7 was recorded during the intestinal digestion phase. All digestions were controlled at 37 °C under stirring at 100 r/min.

The degree of hydrolysis within the gastric and intestinal phases was fitted against a biphasic diffusion model that combines two consecutive steps of first-order kinetics, where the first phase deals with fast diffusion and protein hydrolysis, while the second phase deals with slow or sustained diffusion, using the equation below:

(1) $$y=y_{o}+A_{1}\left(1-e^{-k_{1}t}\right)+A_{2}\left(1-e^{-k_{2}t}\right)$$

where *A*_1_ and *A*_2_ are concentrations of acid or base (µmmol) in the first and second steps, *k*_1_ and *k*_2_ represent the first and second step reaction rate constants (min^−1^), respectively, *y*_0_ is the initial acid/base concentration of the reaction, and t is the digestion time (min).

### 4.13. Statistical Analysis

Independent trials were conducted at different times using fresh samples. Rheological analysis, WHC, gel solubility, and digestion were repeated twice, while textural analysis was repeated three times. Data analysis was performed using SPSS (version 20.0). Tukey’s multiple-comparison test was used to detect significant differences (*p* < 0.05). SigmaPlot 14 (Systat Software Inc., San Jose, CA, USA) was used to create graphs.

## Figures and Tables

**Table 1:** The solubility (mg/mL) of the emulsion-filled gels with a matrix of hybrid soy and whey protein at different ratios in different solvents.

S/W	0/10	3/7	5/5	7/3	10/0
S1-PB	0.41 ± 0.02 a	0.41 ± 0.00 a	0.38 ± 0.2 a	0.32 ± 0.06 b	0.22 ± 0.01 c
S2-Urea	1.38 ± 0.02 a	1.1 ± 0.06 b	0.67 ± 0.13 c	0.63 ± 0.24 c	0.47 ± 0.24 d
S3-SDS	0.36 ± 0.01 c	0.42 ± 0.02 c	2.45 ± 0.22 a	1.00 ± 0.22 b	0.84 ± 0.02 b
S4-β-ME	3.07 ± 0.11 c	2.88 ± 0.14 c	2.99 ± 0.26 c	3.78 ± 0.24 b	4.46 ± 0.21 a

Data that does not share a letter across a row is significantly different (*p* < 0.05).

**Table 2:** Parameters of the biphasic model for enzyme protein hydrolysis and diffusion during simulated gastric and intestinal digestion.

S/W	Order *	Gastric Digestion				Intestinal Digestion			
		y_0_	A/(µmol)	k/(min^−1^)	R^2^	y_1_	A/(µmol)	k/(min^−1^)	R^2^
0/10	1	4.314	219.944	0.413	0.998	13.056	899.542	0.189	0.999
	2		598.370	0.028			1160.749	0.006	
3/7	1	−2.305	439.482	0.141	0.999	23.779	847.574	0.232	0.999
	2		680.575	0.010			855.632	0.027	
5/5	1	−1.150	333.393	0.156	0.998	18.321	901.557	0.264	0.999
	2		6982.328	0.0003			943.325	0.031	
7/3	1	6.381	336.373	0.200	0.999	18.029	1006.730	0.210	0.999
	2		473.272	0.026			679.083	0.021	
10/0	1	−0.265	405.580	0.203	0.999	23.396	724.630	0.269	0.997
	2		365.297	0.019			925.665	0.010	

* Where 1 and 2 indicate the first and second steps of the biphasic model, respectively.

## Data Availability

The data will be made available upon request.
